# The Story of the Insane from Year to Year

**Published:** 1901-01-19

**Authors:** 


### THE STORY OF THE INSANE FROM YEAR TO YEAR.

(Thirteenth Series.)

### THE FIFE AND KINROSS ASYLUM.

During their year, which possesses the curious habit of running from July to July, the asylum numbers increased from 516 patients to 519. There were 83 first admissions, 33 re-admissions, 38 recoveries, 34 discharged relieved, and 41 deaths. The recoveries stand at 31·81 per cent. of the admissions, and the deaths at 5·31 of the average number resident. Of the deaths 21 were caused by cerebral and spinal diseases, 7 by consumption, 1 by pneumonia, and 1 by bronchitis. There was also 1 death from enteric fever. The assigned causes of the insanity in the year's admissions were: moral causes, 10; intemperance in drink, 16; previous attacks, 36; hereditary influence, 47; and in 22 cases no cause was known. Dr. Turnbull points out with much clearness that there is an ebb and flow in the admission rate, but that unfortunately "the general tendency is constantly to rise to a higher average rate"; and he thinks that the accumulation of lunatics in asylums can best be checked "by the discharge of cases who are still of unsound mind, but have improved so far as to be manageable without institutional control." If this is to be done reasonable care must be exercised in choosing the patients for discharge. The matron (Miss Burton), resigned during the year, and was succeeded by Miss Berwick. The committee say they have every reason to be satisfied with the care and attention of Dr. Turnbull. The cost per head per annum was £21 17s.

### THE WEST SUSSEX ASYLUM AT CHICHESTER.

Here an increase of patients from 466 to 479 has to be noted in the year 1899. There were 95 first admissions, 5 re-admissions, 35 recoveries, 15 discharged relieved, and 32 deaths. The recoveries stand at 36·46 per cent. of the admissions, and the deaths at 6·69 per cent. of the average number resident. The recoveries are about the average of similar institutions, but the death-rate is considerably below the average. Of the year's deaths 12 were caused by cerebral and spinal diseases, 4 by phthisis, and 2 by pneumonia. Of the admissions 26 were supposed to owe their insanity to such moral causes as domestic trouble, mental excitement, and religion; while physical causes were represented chiefly by drink (14 cases), by hereditary influence (41), and previous attacks (23). Dr. Kidd informs us that a post-mortem examination took place in every case of death, and that in only one instance was a bedsore discovered—a fact which he says "is practical evidence of good nursing," to which we feel inclined to say "possibly," but by no means "certainly." Dr. Kidd records much useful information about the expense and renewal of electric plant, and of the cost per unit; and he adds that the system of heating the building is a source of great expense; and he arrives at the conclusion—a very sensible one—that "there is a good deal to be said in favour of the old-fashioned open fires in the wards." He might have gone further and said that no system is tolerable without them, although some plans are occasionally useful, and that no man who understands physical science should recommend the *exclusive* use of any hot-air or hot-water system. The Committee say "they are able to report very favourably upon the management of the asylum." The weekly maintenance rate is 11s. 8d. per head; but it does not seem to be set out in tabular form under different headings, as is customary in asylums.

### THE PLYMOUTH BOROUGH ASYLUM.

On January 1st, 1899, this asylum contained 269 patients and at the end of December the number had fallen to 263. There were 81 first admissions, 11 re-admissions, 30 recoveries, 21 discharged relieved, and 29 deaths. The recoveries, calculated on the admissions, were 34·88 per cent., and the deaths, calculated on the average number resident, were 10·74 per cent. We failed to find any table showing the causes of death of those who died in 1899. Of the admissions 12 owed their insanity to various moral causes, 9 to intemperance in drink, and 18 to hereditary influence. Although only eight or nine years old, the asylum is almost full, there being only eighteen vacant beds; and Dr. Bowes points out that the average annual increase of Plymouth patients is twelve, so that in two years' time the beds will all be occupied. Unquestionably Dr. Bowes is right in recommending extension of the asylum, and we should like to add that such extension should be immediately proceeded with, otherwise money will be wasted and much inconvenience caused. The Committee "congratulate Dr. Bowes upon the conspicuous success which has attended the first year of his administration as medical superintendent." The maintenance rate is a fraction under 10s. per head per week.

### THE ENNIS DISTRICT ASYLUM.

On January 1st this asylum contained 376 patients, and at the end of December, 1899, the number had risen to 383. There were 70 first admissions, 77 re-admissions, 39 recoveries, 79 discharged not improved, and 15 deaths. The recoveries calculated on the admissions were 26·5 per cent., and the deaths calculated on the average number resident were 3·9. The recovery rate is below the average, and the death rate is one of the lowest we have had to chronicle. There was not one death from either cerebral or spinal disease, but 9 were from consumption and 3 from other lung affections. Of the year's admissions 21 are supposed to owe their insanity to various moral agencies, and among the physical causes hereditary influence comes first with 42, previous attacks with 22, and intemperance in drink with 6. The asylum is full, and many patients have been discharged to their respective workhouses. By this means some temporary, but very temporary, relief may be obtained, and, as Dr. O'Mara puts it, the patients return again after a short time, and, we should like to add, often deteriorated. It has been practically decided to add another 300 beds to the asylum, and if this be carried out soon it will relieve the pressure for many years to come. The late medical superintendent, (Dr. Gelston), retired on his superannuation allowance "after a long and able service." The annual cost per head was £24 15s. 7d.

